# Reading the ground: Understanding the response of bioelectric microbes to anthropogenic compounds in soil based terrestrial microbial fuel cells

**DOI:** 10.1371/journal.pone.0260528

**Published:** 2021-12-22

**Authors:** Robyn A. Barbato, Robert M. Jones, Michael A. Musty, Scott M. Slone

**Affiliations:** US Army Engineer Research and Development Center Cold Regions Research and Engineering Laboratory, Hanover, NH, United States of America; Universiti Sains Malaysia, MALAYSIA

## Abstract

Electrogenic bacteria produce power in soil based terrestrial microbial fuel cells (tMFCs) by growing on electrodes and transferring electrons released from the breakdown of substrates. The direction and magnitude of voltage production is hypothesized to be dependent on the available substrates. A sensor technology was developed for compounds indicative of anthropological activity by exposing tMFCs to gasoline, petroleum, 2,4-dinitrotoluene, fertilizer, and urea. A machine learning classifier was trained to identify compounds based on the voltage patterns. After 5 to 10 days, the mean voltage stabilized (+/- 0.5 mV). After the entire incubation, voltage ranged from -59.1 mV to 631.8 mV, with the tMFCs containing urea and gasoline producing the highest (624 mV) and lowest (-9 mV) average voltage, respectively. The machine learning algorithm effectively discerned between gasoline, urea, and fertilizer with greater than 94% accuracy, demonstrating that this technology could be successfully operated as an environmental sensor for change detection.

## 1. Introduction

It has been postulated that electrogenic bacteria are found in nearly every ecosystem on the planet; terrestrial environments in particular host a diversity of electrogenic bacteria and substrates. In terrestrial microbial fuel cells (tMFCs) composed of soil, electrogenic bacteria produce electricity through the oxidation of soil organic matter and the reduction of compounds such as sulfate, nitrate, and iron [1, 2]. Examples of known electrogenic bacteria include *Geobacter* sulfurreducens [3], *Rhodoferax ferrireducens* [4], *Shewanella putrefaciens* [5], *Clostridium* spp. [6], and *Bradyrhizobium* spp. [7]. Electrogenic bacteria are able to access substrates and transfer electrons through mechanisms such as nanowires, soluble electron shuttles (e.g. riboflavin), or cytochromes [8]. The anodic microbial community and the anode material influences the electro-activity, electron acceptance by the anode, making them critical components in power generation [9]. Any hindrance in the ability of the electrogenic bacteria to transfer electrons to the electrode (e.g. low surface area, environmental perturbations, methanogens competing with the anode for electrons) typically results in a power differential.

MFCs have been constructed using sediments from aquatic environments [10] or terrestrial systems [7] to serve as alternative sustainable and renewable energy sources. MFCs with marine and wastewater as source materials typically yield higher voltage, ranging from 500 to 800 mV overall [11], while those measured from soil systems are typically lower, ranging from 300 to 400 mV [7]. An ideal design to enhance power production is one that incorporates anoxic conditions at the anode and oxic conditions at the cathode, though many designs have been explored [12].

The flow of electrons is monitored through voltage or current measurements without disturbing the system. Non-destructive monitoring makes the MFC an attractive candidate for biomonitoring, warranting their use as indicators of system health and activity in wastewater systems [11]. More recently, MFCs have been used for bioremediation of contaminants [12] and environmental monitoring of pollutants [13]. Contaminated soil or water is used as the substrate to the MFCs, leading to lower concentrations of the contaminants in the system. Examples of chemicals tested include polyaromatic hydrocarbons [14] and heavy metals [15, 16]. Advantages to using MFCs for remediation and environmental monitoring include that they are low cost, low maintenance, self-sustaining, and respond rapidly [17] which also makes them a promising biosensing technology.

**Fig 1:**
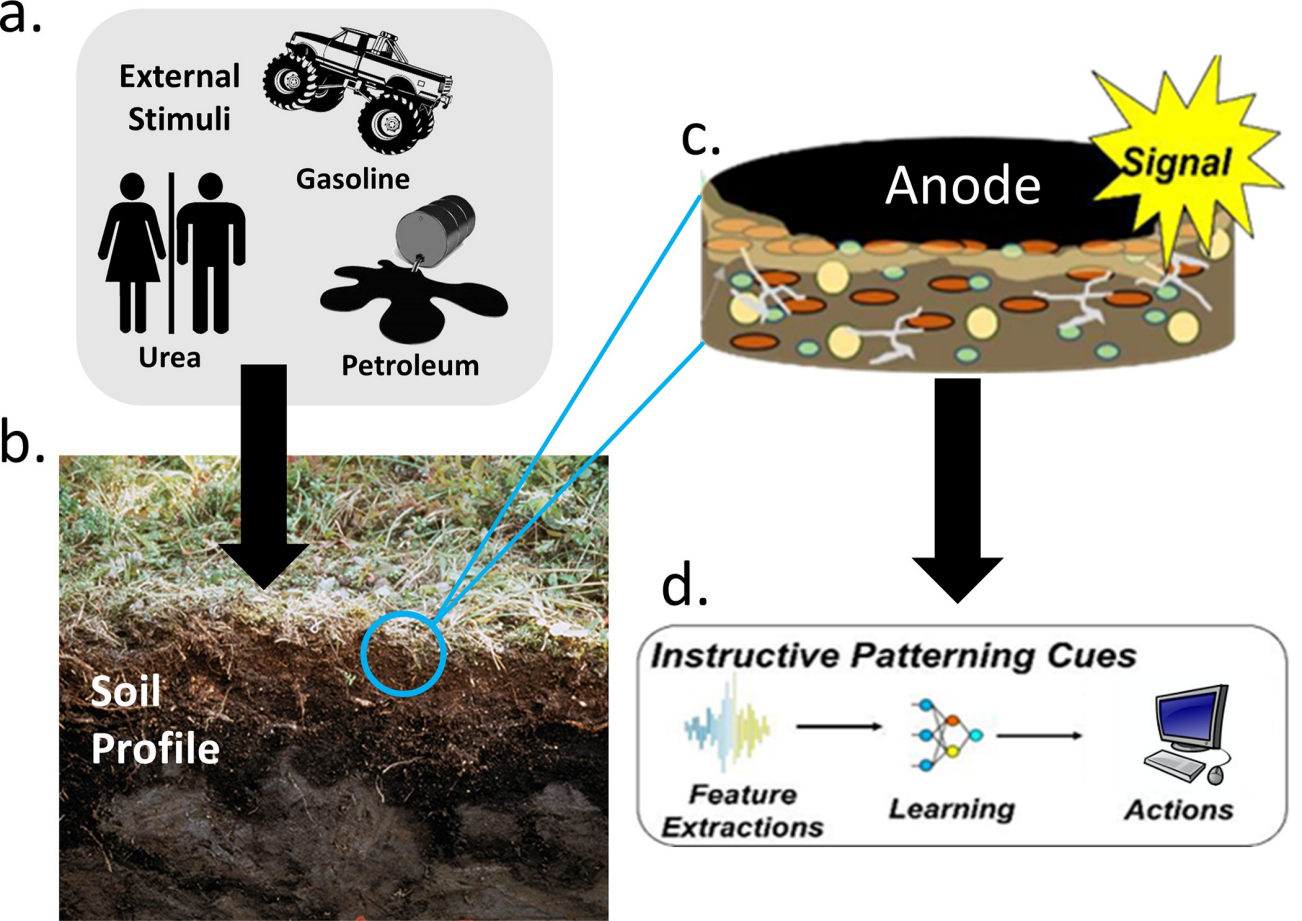
The additional external stimuli may solicit a repeatable response in electrogenic bacteria embedded on electrodes in soil.

Significant advancements have been made regarding microorganisms that contribute to power production, optimization of the design of MFCs, and alternative substrates as sources of inoculum for MFCs. Despite these improvements, a critical research gap remains in understanding voltage changes in tMFCs following the addition of various chemicals and whether machine learning algorithms can effectively classify the AC. The aim of this study was to develop a biologically-based sensor technology that could detect of anthropological compounds (ACs) in soil. The incorporation of ACs into the tMFCs was hypothesized to induce a significant shift in the bioelectrical signals generated by electrogenic soil bacteria (Fig 1). Several ACs, represented as urea, gasoline, petroleum, 2,4-dinitrotoluene, or fertilizer, were added to soil to construct tMFCs. Voltage was measured to determine how external stimuli impacted the tMFCs performance.

## 2. Materials and methods

### 2.1 Microcosm construction and incubation study

**Fig 2:**
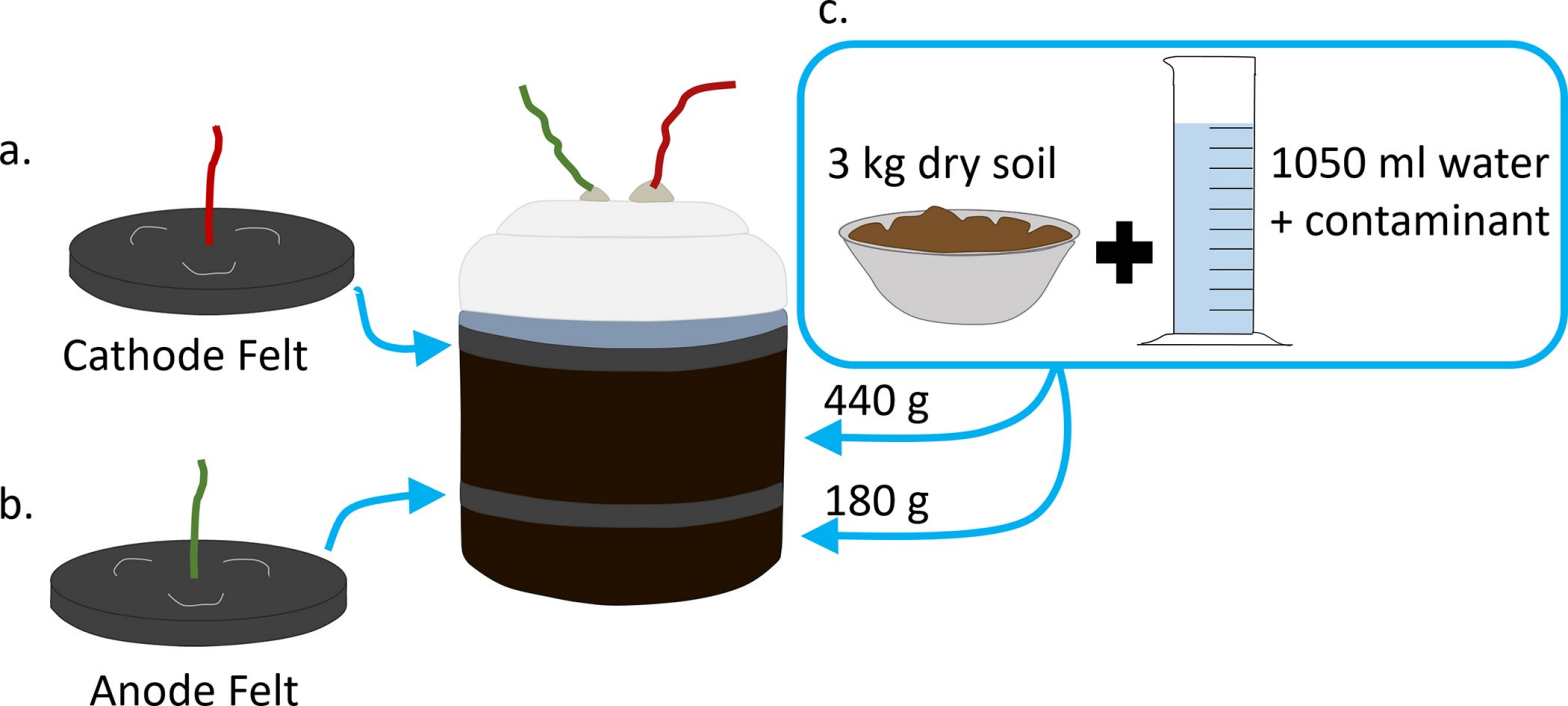
Diagram of microbial fuel cell construction. Wetted soil (with and without contaminant) was distributed below and above the anode before the cathode was placed on top. To reduce the loss of moisture the ports around the wires were sealed with a sticky tack.

A sandy loam soil amended with organic matter was used to construct the tMFCs. Soil particle size distribution was 67.2% sand, 26.3% silt, and 6.5% clay [18]. Soil pH was 7, organic matter content was 3.3% and nitrogen content was 0.2% [18]. For each set of treatments, soil was prepared in batches and then distributed into single chamber microcosms. To achieve a final gravimetric moisture content of 35%, approximately 1,050 mL of liquid (deionized water and the contaminant) was added to 3 kg of dry soil. The final concentrations of contaminants in the soil were 173 μg cm^-3^ 2,4-dinitrotoluene (DNT; CAS 121-14-2; purity 97%, Sigma-Aldrich, St. Louis, MO), 10,266 μg cm^-3^ urea (Bio-Rad Laboratories, Hercules, CA), 2,000 μg cm^-3^ fertilizer (Jack’s 20-20-20 NPK, JR Peters Inc., Allentown, PA), and 25% volume/volume of gasoline or petroleum. For each microcosm, 180 g of wet soil was well mixed and added into a plastic container that was cleaned with 70% ethanol prior to use. Soil was gently tamped to remove air bubbles, achieving an approximate bulk density of 1.3 g cm^-3^. A graphite felt anode was placed on top of the soil (Fig 2). Next, 440 g of wet soil was added on top of the anode. Soil was tamped and a graphite felt cathode was placed on top (Fig 2). The electrode wire holes were sealed with sticky tack to prevent the tMFCs from drying out over time. This process was repeated to achieve six replicate microcosms per treatment. All microcosms were incubated in the dark at 25°C and voltage was measured twice a day for 31 days using a hand-held multimeter (Gardner Bender, Milwaukee, WI). The first voltage measurement occurred 12 hours after the tMFCs were assembled.

### 2.2 Statistical analysis

To maintain a balanced design for the experiment, an observation period between 6 and 31 days of incubation whereby most of the voltage slopes were 0 +/- 0.5 mV was used. Starting from the first day, the voltage values were grouped in time periods of 3, 4, 5, and 6 consecutive measurements which corresponded to 1.5, 2, 2.5, and 3 days, respectively. For each time period, the slope between points was calculated for each replicate and for each treatment, which was used to determine the point of stability. Output of the slopes by point grouping are in S1 File.

Linear mixed effects (LME) models were used to determine differences between treatments over time. The LME models were fit using maximum likelihood estimation (MLE) following Demidenko [19]. The analysis was conducted using the lme4 package [20] in R (Version 3.6.1) [21]. To calculate confidence intervals for model coefficients, the Satterthwaite’s degrees of freedom method was used [22].

Two LME models were used to compare treatment effects. The baseline LME model, which did not group the six treatments independently, was compared with a LME model that included the treatment groupings. To define the baseline model, let *i* = 1,…,*N* with *N* = 36 the number of tMFCs observed in the experiment and let *j* = 1,…,*n*_*i*_ with *n*_*i*_ = 210 the number of voltage measurements for the *i*th tMFC. The baseline model was defined by the equation *y*_*ij*_ = *α*_*i*_+*β*_*i*_*t*_*j*_+*ε*_*ij*_ where *y*_*ij*_ is the voltage (measured in mV) of the *i*th tMFC at time *t*_*j*_ (measured in days since start) and the residual errors *ε*_*ij*_ are assumed to be normally distributed around zero. The LME model also assumes that the regression coefficients *α*_*i*_ and *β*_*i*_ are random and can be expressed by the expressions *α*+*a*_*i*_ and *β*+*b*_*i*_ respectively. The residuals *a*_*i*_ and *b*_*i*_ are assumed to follow a bivariate normal distribution centered at zero. *α* and *β* were the fixed effects of the model whereas *a*_*i*_ and *b*_*i*_ were the random effects (Table 1). The lme4 formula for the baseline model is given by *Voltage*~*Days*+(1|*tMFC*) where *Voltage* indicates the voltage measurement, *Days* indicates the time since the start of observation, and *tMFC* identifies the individual tMFCs.

**Table 1:** Comparison of the baseline linear mixed effects (LME) model (without using treatments) and a LME model using the treatments as a predictor.

	LME baseline model	LME model with treatments
Intercept	177.42 (32.35) ***	339.94 (28.33) ***
Slope	4.48 (0.72) ***	1.73 (1.04)
Gasoline Intercept		-472.09 (40.06) ***
Gasoline Slope		1.65 (1.47)
DNT Intercept		-12.19 (40.06)
DNT Slope		-0.79 (1.47)
Urea Intercept		15.7 (40.06)
Urea Slope		7.08 (1.47) ***
Fertilizer Intercept		-290.22 (40.06) ***
Fertilizer Slope		0.24 (1.47)
Petroleum Intercept		-216.33 (40.06) ***
Petroleum Slope		8.31 (1.47) ***
AIC	13412.63	13187.95
BIC	13443.47	13270.17
*R*^2^ (fixed)	0.03	0.93
*R*^2^ (total)	0.96	0.96

The data used for these models consists of all 36 tMFCs each measured 210 times during the observation period. Intercepts and slopes are model parameters (explained in the methods section) each with a corresponding standard error (in parentheses) and significance indicator (***p<0.001, **p<0.01, *p<0.05). The Akaike information criterion (AIC) values and Bayesian information criterion (BIC) values are included for model comparison. These values indicate that including treatments improves the model. This is reinforced by the likelihood ratio test (not shown in this table) indicating that including the treatments improves the model fit significantly (*p*<0.001). Lastly, the table includes *R*^2^ values indicating how much variation is explained by the fixed and random effects of each model. When the treatments are included in the model nearly all the variation is explained by the fixed effects.

To incorporate the treatments, the same baseline model defined above was used and the model allowed for the treatment groups to influence the random coefficients. This introduced additional fixed effects comparing each treatment to the control. More precisely, the updated lme4 formula was *Voltage*~*Days*+*Treatment*+*Days*:*Treatment*+(1|*tMFC*) where *Treatment* identifies the treatment group and all other variables in the formula are consistent with the baseline model. These additional fixed effects comparing treatment intercepts and slopes to the control were estimated (Table 1). The two LME models were compared using Akaike Information Criterion (AIC) values, Bayesian Information Criterion (BIC) values, *R*^2^ values, and the likelihood ratio test.

Analysis of variance using the aov function as part of the vegan package within R (Version 3.6.1) [21, 23] was performed to calculate significant difference of voltage between treatments at the end of the study. A Tukey’s post hoc tested of honest significant difference using the TukeyHSD function within R [21].

### 2.3 The NM500’s capabilities and internal methods

The NM500 machine-learning computer chip (General Vision Inc., Larkspur, CA) classified ACs based on voltage output by assigning categorical labels to continuous data, such as temperature, chemical content, and voltage, as well as providing a confidence metric [24]. The NM500’s neurons interpreted the results and provided a classification based on how close the parameters are to previously trained values. The NM500 reported the level of agreement among the neurons’ interpretations as a confidence and bins them into the following categories: “Identified” (unanimous agreement amongst the neurons), “Uncertain” (some disagreement), or “Unknown” (insufficient number of neurons fired to make a classification). No classification was given if the confidence was “Unknown” [25].

The NM500 trains using one of two classification methods: K-Nearest Neighbor (KNN) [26] or Radial Basis Function (RBF) [27]. For KNN, a tested input’s classification is defined by its K nearest neighbors among the training inputs, where the majority classification of the K nearest neighbors is the test input’s classification. As all neurons fire in the KNN method, it cannot output a confidence of “Unknown”. The RBF method is similar to KNN, but neurons only fire if their internal weights are within a unit distance of the input parameters. If input parameters are too far from previously seen values, an insufficient number of neurons will fire and the model will produce a confidence of “Unknown” [28].

Interpreters in the model select a classification based on the neuron outputs. “Dominant”, the only interpreter of the K-Nearest Neighbor method, selects the majority classification among the fired neurons as the output. The next is “Best Match”, where the classification of the neuron closest to the input is selected. The third is “Unanimous”, which is a conservative approach similar to “Dominant”, but all “Uncertain” confidence values are instead marked as “Unknown”. The last is “Minimum Consensus”, which is similar to “Unanimous” and “Dominant”, but requires a minimum number of agreeing neurons to provide an output. This minimum number can be adjusted, with larger values making the model increasingly conservative [28].

### 2.4 Input parameters for the machine learning model

To classify the ACs, the main input parameter was the time-dependent voltage of the tMFCs. In order to achieve the number of data points needed to train the machine learning models, data points were spliced down into 2-, 2.5-, and 3-day segments, which allowed the reuse of the same data in multiple smaller segments (see example in Table 2). The amount of training data was increased using this splicing technique. In the 6-point example, where 3 days were used per dataset, the data expanded to 3,672 data points, excluding edge effects and interruptions in the data. The data were prepared using 5- and 4-point groupings, which produced 3,570 and 2,912 data points, respectively. More options were not analyzed in an effort to maintain the long-term fluctuations in voltage that could be vital to classification. The dataset was split into training and testing sets to determine the model’s internal weights and define their accuracy, respectively. Moreover, 20% of the total dataset was placed in the testing set, while the rest were used for training.

**Table 2:** Example of staggering method to generate data for the machine learning model.

Timestamp	Original Dataset (Control-R1)	Data Grouping				
		1	2	3	4	5
Day 6 AM	253.7	253.7				
Day 6 PM	245.1	245.1	245.1			
Day 7 AM	244.4	244.4	244.4	244.4		
Day 7 PM	259.9	259.9	259.9	259.9	259.9	
Day 8 AM	259.0	259.0	259.0	259.0	259.0	259.0
Day 8 PM	250.4	250.4	250.4	250.4	250.4	250.4
Day 9 AM	241.9		241.9	241.9	241.9	241.9
Day 9 PM	252.6			252.6	252.6	252.6
Day 10 AM	250.2				250.2	250.2
Day 10 PM	253.4					253.4

Example data is from Day 6–10 of Control Replicate R1. By pairing the original 10 data points into groups of 6, we can produce 5 synthetic datasets while still preserving the internal voltage fluctuation. Each number represents 5 days of voltage from one of the replicates.

To identify the influence of standardization of input values on model accuracy, data were adjusted using z-score standardization with a mean of 0 and a standard deviation of 1. Several methods of grouping the data for standardization were considered, such as the entire dataset, each AC separately, or adjusting the entire dataset based on control’s mean and standard deviation rather than the entire dataset’s mean and standard deviation. This last method would not necessarily produce a dataset with a mean of 0 and a standard deviation of 1, but instead would adjust the values with reference to the control potentially making AC influences more distinct. Besides the raw unstandardized dataset, the NM500 was trained on three standardization grouping methods (all, each AC, and control).

A total of four model settings were tested with the NM500: size of point groupings (i.e. 4–6 pts), standardization (i.e. raw, all, AC-specific, control-specific), method (i.e. KNN, RBF), and interpreter (i.e. “Dominant”, “Best Match”, “Unanimous”, and “Minimum Consensus”). Due to KNN only using the “Dominant” Interpreter, the K value was set to 3, 4, 5, 10, and 15 nearest neighbors, overall producing 120 models between all settings considered. The ten best performing models with the highest accuracies for “General” and “Identified Accuracy” were identified (S1 File). Models with “Unknowns” greater than 50% were not considered because they were poor classifiers and classifications with less than 25% considered “Identified” were removed because they lacked sufficient confidence.

## 3. Results and discussion

### 3.1 Anthropogenic compound impacts voltage patterns in tMFCs

**Fig 3:**
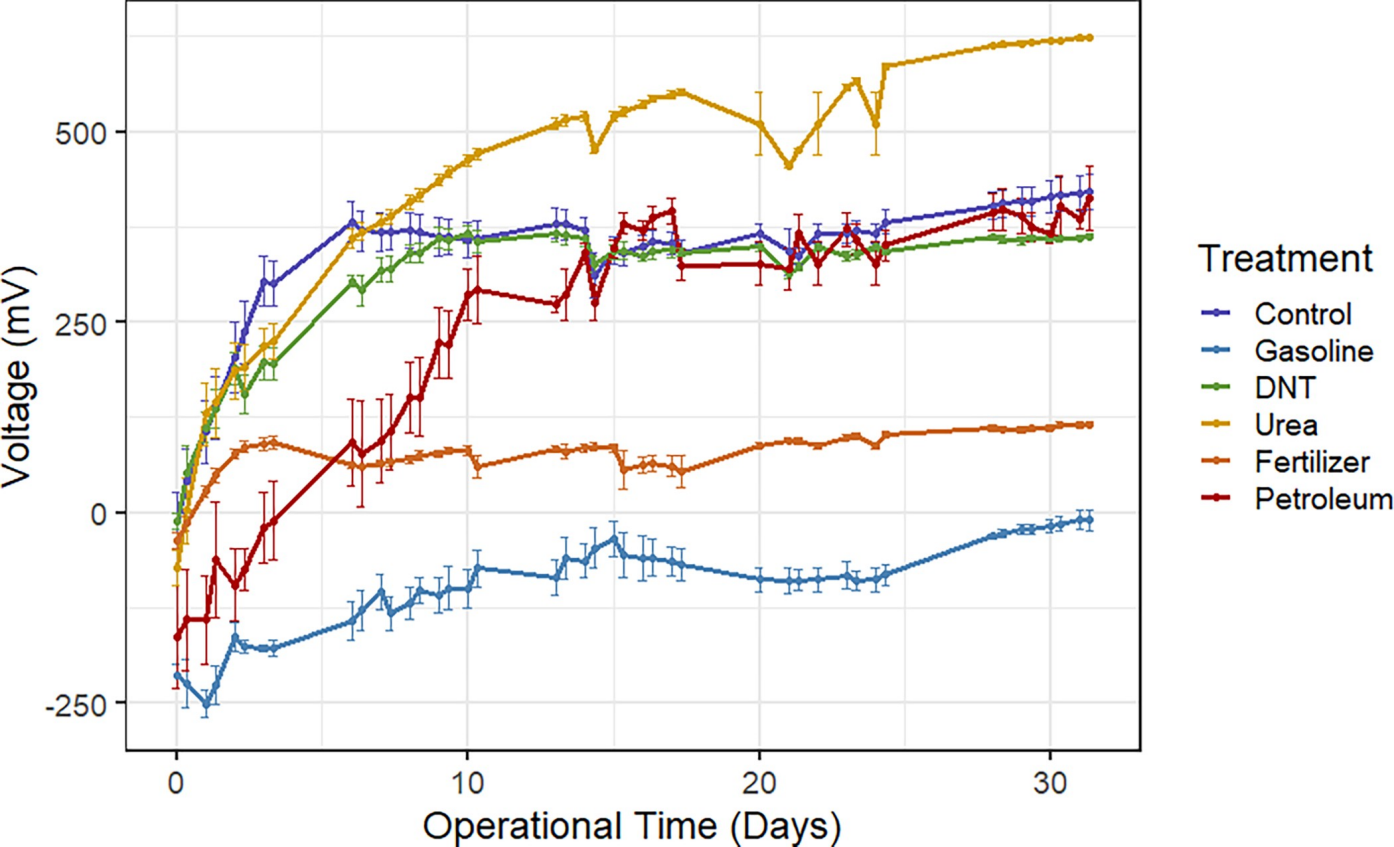
Evolution of voltage from tMFCs for the duration of the incubation. Lines indicate mean voltage. Bars indicate standard error of n = 6 replicates.

**Fig 4:**
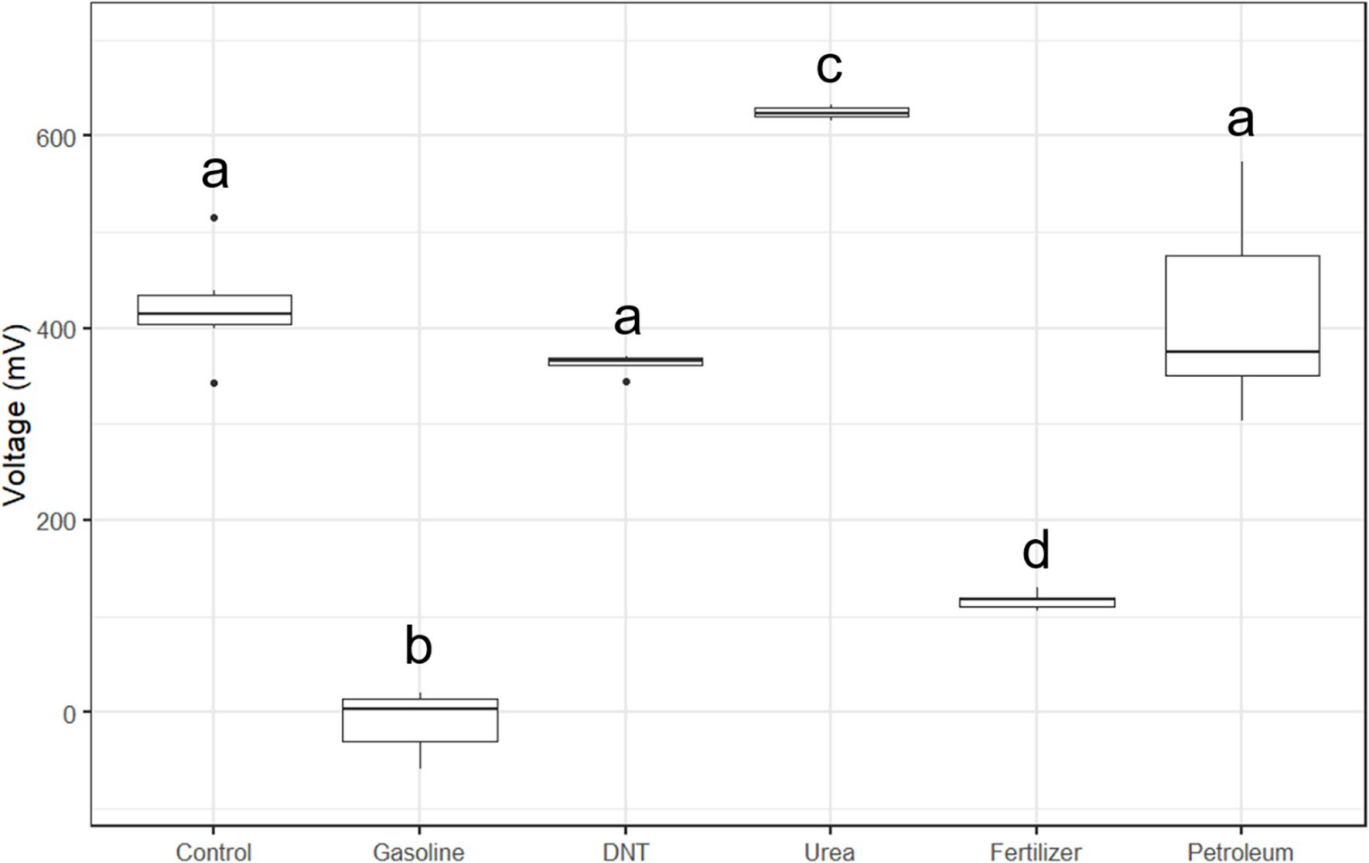
Boxplots of end state voltage data across the six treatments (control, gasoline, DNT, urea, fertilizer, or petroleum). Each treatment has sample size n = 6.

The first objective was to determine if tMFCs mixed with different ACs influenced voltage output compared to control tMFCs. In the treated and control tMFCs, the average voltage across the six replicates increased initially and then stabilized (slope < 0.5 mV) between 5 and 10 days of incubation (Fig 3). The average voltage varied among treatments, with urea tMFCs exhibiting the highest average voltage of 287 mV over the first ten days of incubation period and gasoline exhibiting the lowest average voltage of 150 mV during that time period (Fig 3). At day 10, the average voltage outputs for urea and gasoline were 470 mV and –73 mV, respectively (Fig 3). Interestingly, the petroleum and gasoline tMFCs transmitted negative average voltage values after two days of incubation, with the gasoline treatment remaining negative for the entire study period (Fig 3). Though the petroleum tMFCs exhibited an initial lag in voltage performance, they had similar average voltage outputs as the control and DNT tMFCs after 15 days of incubation (Fig 3). Of the treatments tested, the fertilizer tMFCs had the smallest range in voltage from -36 mV to 116 mV over the incubation period, while petroleum had the greatest range in average voltage, from -163 mV to 413 mV (Fig 3). At the end of the incubation period, the average voltage from the urea tMFCs was 624 mV, which was significantly (*p* < 0.0001) higher than that measured from the control microcosms at 421 mV (Fig 4). Urea serves as a nitrogen source for bioelectric bacteria, resulting in a higher voltage output, as evidenced in tMFCs with compost as a urea source [29]. In nitrogen-limited soils, urea has been shown to increase soil productivity [30], though the long-term effects of adding urea may result in a nitrogen deficiency in the soil system [31].

Interestingly, the end state average voltage from the petroleum and DNT tMFCs were not significantly different from the control tMFCs (Fig 4). The addition of either of these compounds did not negatively impact the production of electrons by bioelectric microorganisms even though they are toxic to soil microorganisms and plants [32, 33]. Other studies using MFCs to remediate sludge [34] or sediment [35] contaminated with total petroleum hydrocarbons observed voltage values of 49 mV or 190 mV, respectively, which are nearly a quarter or half of the voltage output we observed in the petroleum tMFCs from this study.

Average voltage measurements from the gasoline and fertilizer tMFCs were significantly (*p* < 0.0001) lower than those measured in the control microcosms (Fig 4), indicating that those two chemicals impeded either the colonization of bioelectric bacteria on the anode surface or perhaps limited the production or transfer of electrons to the anode. Gasoline, a derivative of petroleum that is a common pollution source world-wide, had a negative impact on voltage production as compared to petroleum alone. The vapors found in gasoline in the form of benzene, a known carcinogen, toluene, ethylbenzene, and xylene have been shown to reduce soil activity and limit microbial resilience [36]. The low voltage output from the fertilizer tMFCs is surprising because the fertilizer is composed of 10.1% urea, yet the voltage output from the fertilizer tMFCs was significantly lower than that of the urea tMFCs (Fig 4). However, the end state average voltage for the fertilizer tMFCs (i.e. 116 mV) was higher than found in a study in which fertilizer was added to wastewater MFCs (67.5 mV) [37].

### 3.2 Application of linear mixed effects (LME) models to analyze AC effects over the observation period

The second objective was to investigate if the effects of the ACs could be distinguished by the voltage output over the observed time. For this objective, an observation period starting on day six and ending on day 31 was chosen. Two LME models were compared to determine if the voltage output from the tMFCs was significantly different between treatments. The baseline model compared the 36 tMFCs individually and the second model incorporated the treatments. A likelihood ratio test comparing the two models shows that the model with the treatment information fit significantly better (*p* < 0.001). This improvement in model fit was also confirmed by the AIC and BIC values (Table 1). Moreover, the *R*^2^ values in Table 1 show that incorporating the fixed effects of the treatment groups increases the explained variation from 0.03 (for the baseline model) to 0.93. The baseline model, which treated each tMFC individually, has two fixed effects. The intercept estimate is 177.42 and the slope estimate is 4.48 with standard errors 32.35 and 0.72, respectively meaning that the 36 tMFCs had a voltage output of approximately 177.42 mV at the beginning of the observation period and the voltage increased by approximately 4.48 mV each day.

In the second model the intercept and slope estimates of each treatment group were compared to the intercept and slope estimates of the control. For example, the control intercept estimate is 339.94 (with standard error 28.33) and the gasoline intercept estimate is -472.09 (with standard error 40.06). Therefore, the gasoline intercept was 472.09 mV lower than the control intercept. The significance indicator for the gasoline intercept indicates that the intercept for gasoline differs significantly (*p* < 0.001) from that of the control (i.e. that the coefficient for their difference is significantly nonzero). For the other intercept coefficients, gasoline, fertilizer, and petroleum differed from the control intercept while DNT and urea did not. Similarly, for the slope comparisons, urea and petroleum had significantly different slopes compared to the control while gasoline, DNT, and fertilizer did not. This indicates that the voltages for urea and petroleum changed at a different rate compared to the control. One note of caution is that the significant slope of petroleum is due to the choice of observation period (see Fig 3). The positive slope for urea agrees with the findings in Magotra et al. [29] and Sun et al. [31] suggesting that this compound increases voltage output.

LME models can also be used to analyze the variability of individual tMFCs within a group. The overall variability of the 36 tMFCs together can be measured by the standard errors of the parameter estimates for the baseline model in Table 1. The standard errors are 32.35 and 0.72 for intercept and slope, respectively. To analyze the variability of tMFCs within each treatment we use the same baseline model separately fit for each treatment. The standard errors in each of these models measure the variability between tMFCs from the same treatment group. These computations are summarized in Table 3; fertilizer and urea had the lowest variability and petroleum and gasoline had the highest.

**Table 3:** This table contains LME model parameters for six LME models (defined in the methods section).

	Control	Gasoline	DNT	Urea	Fertilizer	Petroleum
Intercept	339.94	-132.15	327.75	355.64	49.72	123.61
	(24.78)	(29.5)	(17.56)	(11.22)	(4.34)	(49.98)
	***	***	***	***	***	*
Slope	1.73 (0.7)	3.38 (0.79)	0.93 (0.68)	8.81 (0.41)	1.97 (0.18)	10.04 (2.04)
	*	**		***	***	**

For each of the six treatments, the estimated intercepts and slopes of the LME model are given. In parentheses is the standard error associated to each estimate along with the corresponding significance indicator ((***p<0.001, **p<0.01, *p<0.05). These standard errors measure the variability of voltage output within a given treatment. This shows that fertilizer and urea are the least variable treatments and petroleum and gasoline are the most variable.

### 3.3 Application of a machine learning model to effectively classify ACs

The third and final objective was to test whether machine learning models could effectively distinguish between the treated tMFCs based on voltage output. The RBF method had the highest accuracy of 96.6% for 6pt-All-RBF-”Unanimous” model settings, while the KNN methods had at best an accuracy of 80.4% for 4pt-All-KNN-5-”Dominant” model settings (Table 4). While KNN is a commonly used method^38-39^, it is not recommended for this dataset due to the poor performance compared to RBF. Standardizations that were AC- or control-specific are also not recommended, as the highest “General Accuracy” of either was 82.9% for 6pt-control-RBF-”Unanimous” at 82.9% (S1 File). Standardizing across all datasets improved accuracy, as 7 of the 10 best performing models had datasets where all replicates were standardized, but one of the models using raw data had 94.7% accuracy. In terms of interpreters, there was no clear best option, as three of the four were equally present in the top 10, with “Unanimous” having the most examples (Table 4). “Minimum Consensus” was not present anywhere, as all models using it had greater than 50% of their classifications being “Unknown”. To maximize “General Accuracy”, it is recommended to use the Radial Basis Function and to standardize across all datasets.

**Table 4:** Machine learning model results for “General Accuracy”.

Model Settings				Correct		Incorrect		“Unknown”	Accuracy	
#pts	Standard.	Method	Interpreter	ID	Unclear	ID	Unclear		All	ID
**6pt**	**All**	**RBF**	**U**	**69.1**		**2.4**		**28.5**	**96.6**	**96.6**
**6pt**	**All**	**RBF**	**BM**	**69.1**	**4.1**	**2.4**	**0.8**	**23.6**	**95.7**	**96.6**
**6pt**	**All**	**RBF**	**D**	**69.1**	**3.3**	**2.4**	**1.6**	**23.6**	**94.7**	**96.6**
**4pt**	**Raw**	**RBF**	**U**	**70.1**		**4.2**		**25.8**	**94.4**	**94.4**
**4pt**	**All**	**RBF**	**U**	**70.1**		**4.2**		**25.8**	**94.4**	**94.4**
5pt	All	RBF	U	72.7		6.3		21.0	92.0	92.0
4pt	All	RBF	D	70.1	7.2	4.2	3.6	15.0	90.9	94.4
5pt	Raw	RBF	U	72.7		7.7		19.6	90.4	90.4
4pt	All	RBF	BM	70.1	6.6	4.2	4.2	15.0	90.1	94.4
6pt	Raw	RBF	BM	68.3	8.1	8.9	0.8	13.8	88.7	88.4

Bold Models are shared with Table 5. Numbers represent percentages. For Interpreter, U is defined as “Unanimous”, BM as “Best Match”, and D as “Dominant”.

The second and third highest identified accuracies came from models using the KNN method, and in fact had perfect accuracies, a consequence of all “Identified” classifications being correct (Table 5). Additionally, the raw data produced a higher “Identified Accuracy” than all standardization methods. For interpreters, “Dominant” had the most examples in the top 10, but “Unanimous” had the highest accuracy (Table 5). To maximize “Identified Accuracy”, either method is recommended, and data could be left as is or standardized across the entire dataset.

**Table 5:** Machine learning model results for “Identified Accuracy”.

Model Settings				Correct		Incorrect		“Unknown”	Accuracy	
#pts	Standard.	Method	Interpreter	ID	Unclear	ID	Unclear		All	ID
6pt	Raw	RBF	U	68.3			8.9	22.8	88.4	100
4pt	Raw	KNN-3	D	25.2	52.7		22.2		77.8	100
5pt	Raw	KNN-3	D	29.3	49.7	0.6	20.4		79.0	98.0
**6pt**	**All**	**RBF**	**U**	**69.1**		**2.4**		**28.5**	**96.6**	**96.6**
**6pt**	**All**	**RBF**	**BM**	**69.1**	**4.1**	**2.4**	**0.8**	**23.6**	**95.7**	**96.6**
**6pt**	**All**	**RBF**	**D**	**69.1**	**3.3**	**2.4**	**1.6**	**23.6**	**94.7**	**96.6**
6pt	All	KNN-3	D	32.5	43.1	1.6	22.8		75.6	95.2
**4pt**	**Raw**	**RBF**	**U**	**70.1**		**4.2**		**25.8**	**94.4**	**94.4**
**4pt**	**All**	**RBF**	**U**	**70.1**		**4.2**		**25.8**	**94.4**	**94.4**
4pt	All	RBF	D	70.1	7.2	4.2	3.6	15.0	90.9	94.4

Bold Models are shared with Table 4. Numbers represent percentages. For Interpreter, U is defined as “Unanimous”, BM as “Best Match”, and D as “Dominant”.

**Fig 5:**
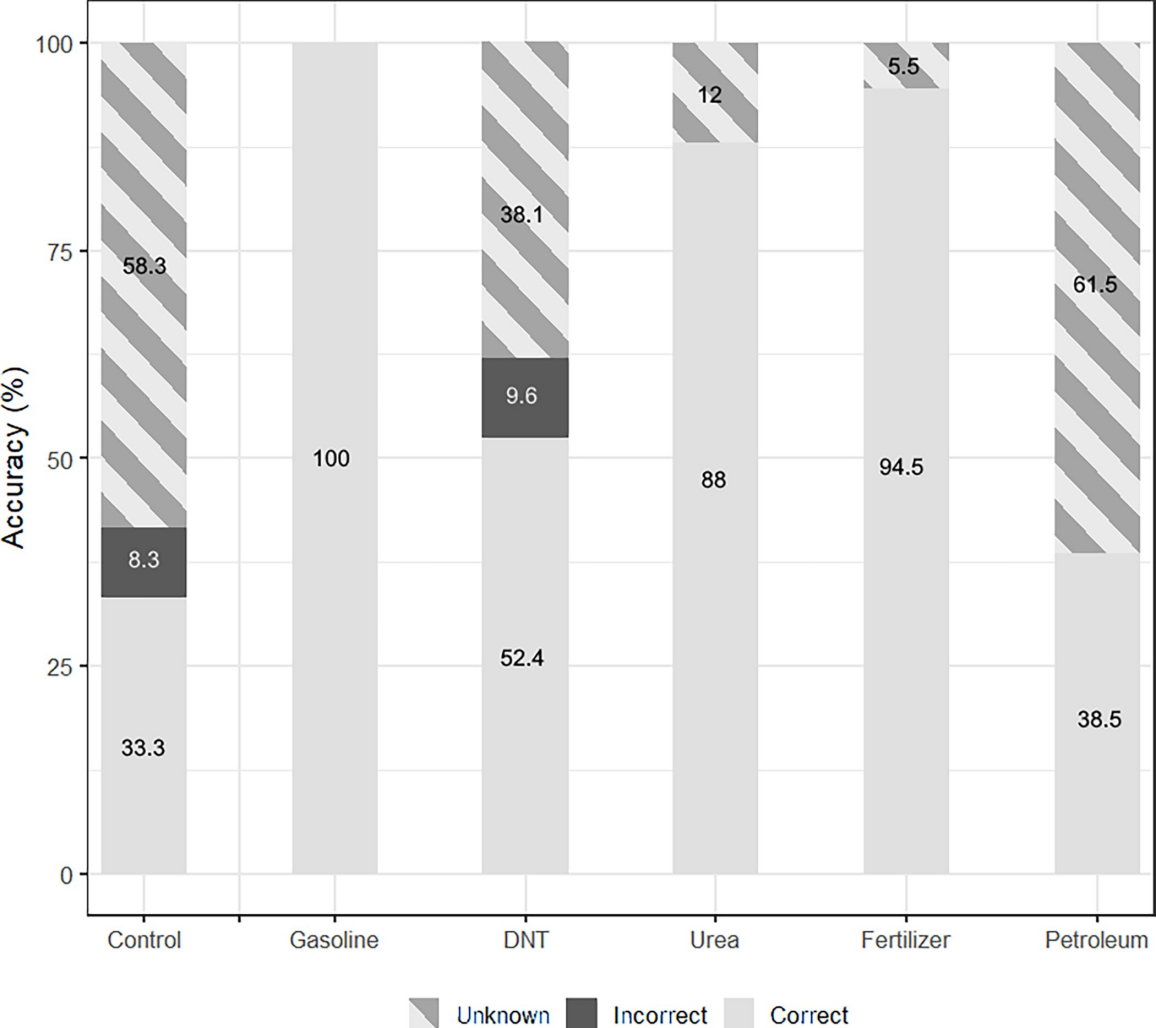
NM500 interpretation of anthropogenic compounds, with all compounds tested. Method used is 6-point grouping with Radial Basis function and “Unanimous” interpreter on standardized data. All answers are marked as “Identified”.

**Fig 6:**
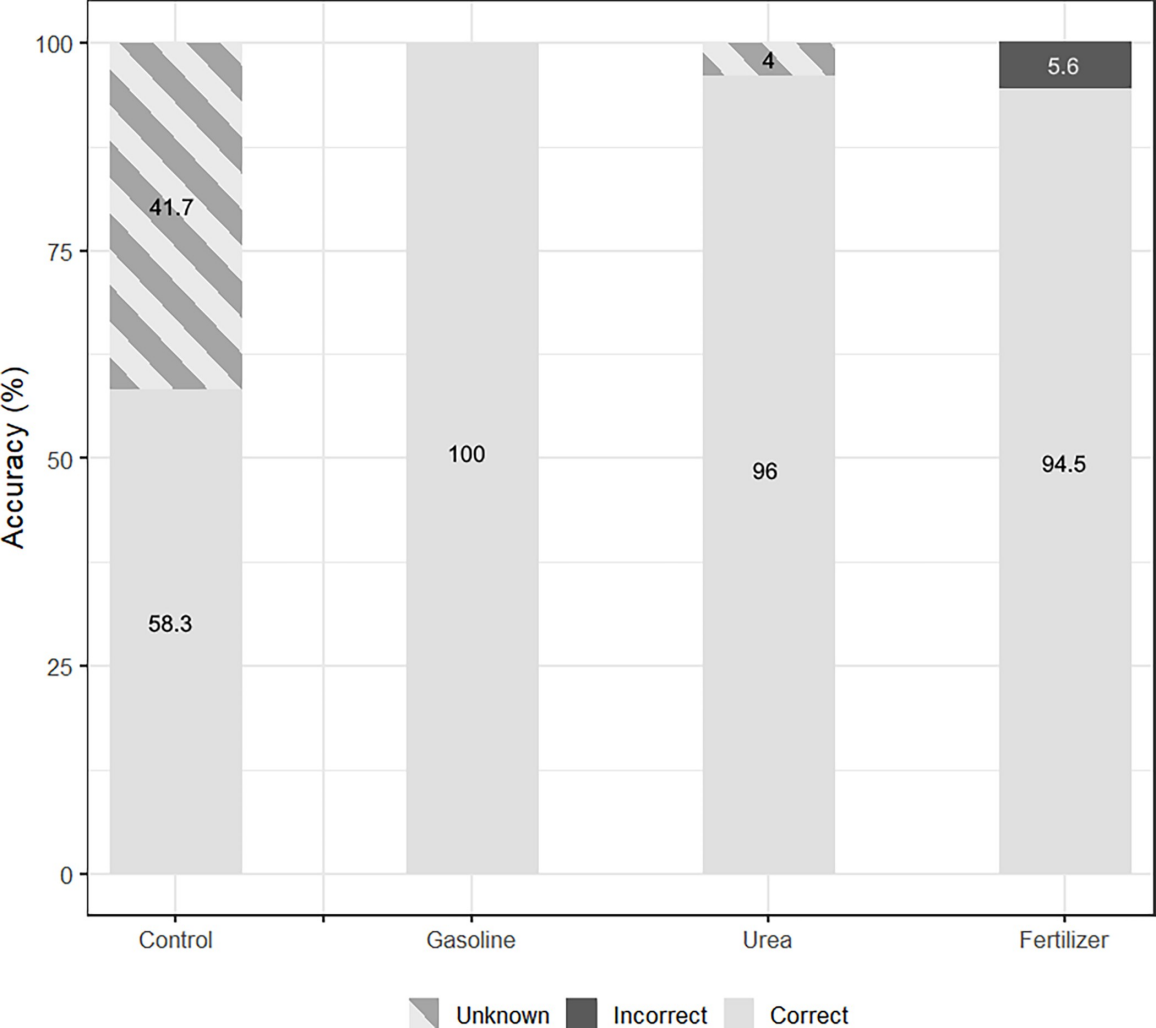
NM500 interpretation of anthropogenic compounds, with only the top 3 most accurate compounds from Fig 5 used for training. Method used is 6-point grouping with Radial Basis function and “Unanimous” interpreter on standardized data. All answers are marked as “Identified”.

Accuracy between model types was tested for each AC (Fig 5). For the model with the highest “General Accuracy”, 6pt-All-RBF-“Unanimous”, the only incorrect predictions were made for the control condition and DNT, with accuracies of 80.1% and 84.6% respectively (Fig 5). There were also issues with high percentages of”Unknowns”, for both the control and DNT; also petroleum, which had the highest percent of classifications marked “Unknown” overall at 61.5%. Incorrect designations tended to be among each other, such as a voltage from a DNT replicate being incorrectly classified as petroleum. This is in agreement with the LME model, as the control and DNT had similar statistical parameters. When the DNT and petroleum datasets were removed, reducing the classifications to between control, gasoline, urea, and fertilizer, the overall accuracy increased (Fig 6). There were fewer answers marked “Incorrect”, and control’s accuracy increased from 80.1% to 100%. This increase in control’s accuracy is expected, as all previous errors were due to misclassifying results as DNT and petroleum. DNT’s intercept and slope did not differ significantly from the control’s, which could explain the issues in its classification. Petroleum, however, differed from the control’s intercept and slope, although its significant slope change was likely due to the observation window. Further testing and statistical analysis would help to explain this and identify if LME models are a useful predictor of machine learning chip efficacy.

Overall, the best results came from the use of 6pt groupings, the RBF method, and the “Unanimous” interpreter (Tables 4 and 5). Standardization influenced accuracy such that “General Accuracy” increased to 96.6%, but raw data had a perfect “Identified Accuracy”. It is recommended in the future to maintain the above grouping selection, training method, and interpreter, but the standardization of data requires further investigation to determine whether it is beneficial. In terms of accuracy, the model also effectively detected tMFCs with gasoline, urea, and fertilizer, but had difficulty differentiating between the control, DNT, and petroleum tMFCs.

The machine-learning algorithm had notable success in the detection of several ACs, getting accuracies above 80% for gasoline, urea, and fertilizer, which increased to above 90% when the machine-learning algorithm was trained only to compare these to the control (Fig 6). This is in agreement with the statistical analysis, where these three ACs had the most distinct voltage responses compared to the control, and DNT and petroleum had similar responses both to each other and to the control (Figs 5 and 6). Visual inspection of the voltage evolution (Fig 3) confirmed this discrepancy because the voltage output from control, DNT, and petroleum tMFCs from day 10 to the end of the incubation behaved similarly. It was found that by removing these two ACs, the overall accuracy of the other four classifications improved (Fig 6). This suggests the most suitable application for the machine learning algorithms tested would be in the detection of gasoline, urea, and fertilizer. While there would be some accuracy in petroleum detection, it would be only in a third of all actual cases, with most detections being marked as “Unknown” due to low confidence.

To maximize “General Accuracy” (Table 4), it was recommended to use the RBF, but the KNN method had some examples of high “Identified Accuracy” (Table 5). KNN is one of the simplest machine learning algorithms [38], and the RBF is an improvement on it that uses the distance between input and trained datasets to better determine confidence and provide “Unknown” confidences [39]. This addition appeared to improve the models’ accuracy, as the 10 best models that had “Unknown” classifications had reduced Unclear outputs, regardless of if they were Correct or Incorrect. This reduction of Unclear outputs meant fewer incorrect answers. An “Unknown” answer, while it does not have an output, does avoid false positives/negatives in detection, and would in practice be similar to output from tMFCs voltage response being unclear as to the presence of an AC. When DNT and petroleum were removed, nearly all of the remaining”Unknowns” were from the controls (Fig 6). This again supports the findings from the statistical analysis, as control’s voltage distribution was closest to DNT and petroleum, but had a wider range of acceptable values (Fig 4). This explains the output of the machine-learning algorithms, as many of the control results were not distinctive and thus the models had low confidence in their classification. However, gasoline, urea, and fertilizer had results that were distinctive and could be “Identified” with very high accuracy. This connection between statistical results and the model’s output indicates that one could be used to infer the other.

This inference could potentially be used to determine the applicability of chip-based machine- learning algorithms. If there is not a statistically significant difference between two voltage distributions, it is unlikely the algorithm will be able to distinguish them with any reasonable success. However, this interpretation may be dependent on the frequency of data acquisition. As results were taken twice a day, the model was likely not affected by hourly fluctuations in the voltage, since standardization by the control or compound specific datasets failed to produce any significantly accurate models. This fluctuation could be distinctive of specific ACs, therefore it is recommended to use a higher frequency of testing in the future to verify its significance, which will help to validate if statistical analysis is a good indicator of machine-learning applicability. Since the voltage readings are relatively low, practical considerations of field conditions and their effects on voltage measurements would bring more confidence to this technology in real-world applications.

## 4. Conclusion

MFCs are promising biosensors to effectively detect compounds that have been added to soil. In this study, the presence of ACs either influenced the trajectory and/or the final voltage produced by electrogenic bacteria during the incubation period. tMFCs with urea produced the highest voltage of 624 mV whereas those in the presence of gasoline had the lowest voltage that nearly reached 0 mV. tMFCs with added gasoline, urea, and fertilizer were significantly (*p* < 0.0001) different from the control tMFCs. When the treatments were included in the linear mixed effects model, nearly all of the variation was explained by the fixed effects. In accordance with the linear mixed effects model results, the radial basis function and unanimous interpreter more effectively classified the gasoline, urea, and fertilizer treatments, with accuracies of 100%, 88%, and 94.5%, respectively. Together, these results show that 1) select ACs significantly shifted voltage produced by bioelectric microorganisms and 2) linear mixed effects models, along with machine learning classifiers effectively characterized a subset of the ACs tested, warranting strong consideration to use tMFCs as biosensors for environmental monitoring. Questions remain regarding the concurrent influence of environmental variables such as precipitation and the presence of ACs. Future work should include evaluating whether the machine learning algorithms effectively classify treatments under a variety of environmental conditions and evaluating the microbial ecology on the electrodes. Additionally, the influence of an AC on an established tMFC (i.e. the spill of a chemical on the soil impregnated with the electrodes) would be more reflective of the application of this technology in the field.

## Supporting information

S1 File Machine learning model parameters tested and determination of the observation window. Values are listed in descending order by “General Accuracy”. All Values are percentages. Blank values imply 0%. AC is defined as standardization by anthropogenic compounds. RBF is defined as Radial Basis Function. KNN-# is defined as K-Nearest Neighbor Method where # is the K-value. For Interpreter, U is defined as “Unanimous”, BM as “Best Match”, D as “Dominant”, and MC-# is defined as the “Minimum Consensus” Interpreter where # is the minimum consensus. N/A means the model had no results for this category. Model Unsuccessful means there were insufficient correct classifications to train the model. To determine the observation window, data points were grouped in 4 (a), 5 (b), and 6 (c) point groups. X axis indicates group number i.e. the first group of four points, second group of four points, etc, Y axis is the slope of the four consecutive points, shape represents the replicate.
(DOCX)
