# Supplementary material for: Reading the ground: Understanding the response of bioelectric microbes to anthropogenic compounds in soil based terrestrial microbial fuel cells
Source: PLoS One. 2021 Dec 22;16(12):e0260528. doi: 10.1371/journal.pone.0260528 (PMC8694411; doi:10.1371/journal.pone.0260528)

## Supporting Information

**S1 File. Machine learning model parameters tested and determination of the observation window.** Values are listed in descending order by “General Accuracy”. All Values are percentages. Blank values imply 0 %. AC is defined as standardization by anthropogenic compounds. RBF is defined as Radial Basis Function. KNN-# is defined as K-Nearest Neighbor Method where # is the K-value. For Interpreter, U is defined as “Unanimous”, BM as “Best Match”, D as “Dominant”, and MC-# is defined as the “Minimum Consensus” Interpreter where # is the minimum consensus. N/A means the model had no results for this category. Model Unsuccessful means there were insufficient correct classifications to train the model. To determine the observation window, data points were grouped in 4 (a), 5 (b), and 6 (c) point groups. X axis indicates group number i.e. the first group of four points, second group of four points, etc, Y axis is the slope of the four consecutive points, shape represents the replicate.

| Model Settings |  |  |  | Correct | | Incorrect | | “Unknown” | Accuracy | |
| --- | --- | --- | --- | --- | --- | --- | --- | --- | --- | --- |
| #pts | Standard. | Method | Interpreter | ID | Unclear | ID | Unclear |  | All | ID |
| 4pt | Raw | RBF | MC-2 | 28.14 | 1.2 |  |  | 70.66 | 100 | 100 |
| 4pt | All | RBF | MC-2 | 25.15 | 1.2 |  |  | 73.65 | 100 | 100 |
| 6pt | All | RBF | MC-3 | 7.32 |  |  |  | 92.68 | 100 | 100 |
| 4pt | Raw | RBF | MC-3 | 2.4 |  |  |  | 97.6 | 100 | 100 |
| 4pt | All | RBF | MC-3 | 1.8 | 0.6 |  |  | 97.6 | 100 | 100 |
| 6pt | All | RBF | MC-2 | 23.58 |  |  | 0.81 | 75.61 | 96.7 | 100 |
| 6pt | All | RBF | U | 69.11 |  | 2.44 |  | 28.46 | 96.6 | 96.6 |
| 6pt | All | RBF | BM | 69.11 | 4.07 | 2.44 | 0.81 | 23.58 | 95.7 | 96.6 |
| 6pt | All | RBF | D | 69.11 | 3.25 | 2.44 | 1.63 | 23.58 | 94.7 | 96.6 |
| 4pt | Raw | RBF | U | 70.06 |  | 4.19 |  | 25.75 | 94.4 | 94.4 |
| 4pt | All | RBF | U | 70.06 |  | 4.19 |  | 25.75 | 94.4 | 94.4 |
| 5pt | All | RBF | U | 72.73 |  | 6.29 |  | 20.98 | 92 | 92 |
| 5pt | Raw | RBF | MC-2 | 19.58 | 2.1 | 1.4 | 0.7 | 76.22 | 91.9 | 93.3 |
| 5pt | All | RBF | MC-2 | 18.88 | 2.1 | 1.4 | 0.7 | 76.92 | 90.9 | 93.1 |
| 4pt | All | RBF | D | 70.06 | 7.19 | 4.19 | 3.59 | 14.97 | 90.9 | 94.4 |
| 5pt | Raw | RBF | U | 72.73 |  | 7.69 |  | 19.58 | 90.4 | 90.4 |
| 4pt | All | RBF | BM | 70.06 | 6.59 | 4.19 | 4.19 | 14.97 | 90.1 | 94.4 |
| 6pt | Raw | RBF | MC-3 | 6.5 | 0.81 | 0.81 |  | 91.87 | 90 | 88.9 |
| 6pt | Raw | RBF | BM | 68.29 | 8.13 | 8.94 | 0.81 | 13.82 | 88.7 | 88.4 |
| 5pt | All | RBF | BM | 72.73 | 7.69 | 6.29 | 4.2 | 9.09 | 88.5 | 92 |
| 5pt | All | RBF | D | 72.73 | 7.69 | 6.29 | 4.2 | 9.09 | 88.5 | 92 |
| 6pt | Raw | RBF | U | 68.29 |  |  | 8.94 | 22.76 | 88.4 | 100 |
| 4pt | Raw | RBF | D | 70.06 | 6.59 | 4.19 | 5.99 | 13.17 | 88.3 | 94.4 |
| 6pt | Raw | RBF | MC-2 | 20.33 | 1.63 | 2.44 | 0.81 | 74.8 | 88.1 | 89.3 |
| 6pt | Raw | RBF | D | 67.48 | 8.94 | 9.76 | 0.81 | 13.01 | 87.8 | 87.4 |
| 5pt | Raw | RBF | D | 72.73 | 7.69 | 7.69 | 3.5 | 8.39 | 87.8 | 90.4 |
| 5pt | Control | RBF | MC-2 | 27.97 | 2.1 | 3.5 | 0.7 | 65.73 | 87.7 | 88.9 |
| 4pt | Raw | RBF | BM | 70.06 | 5.39 | 4.19 | 7.19 | 13.17 | 86.9 | 94.4 |
| 5pt | Raw | RBF | BM | 72.73 | 6.29 | 7.69 | 4.9 | 8.39 | 86.3 | 90.4 |
| 5pt | Raw | RBF | MC-3 | 4.2 |  | 0.7 |  | 95.1 | 85.7 | 85.7 |
| 6pt | Control | RBF | U | 70.73 |  | 14.63 |  | 14.63 | 82.9 | 82.9 |
| 6pt | Control | RBF | BM | 70.73 | 7.32 | 14.63 | 1.63 | 5.69 | 82.8 | 82.9 |
| 6pt | Control | RBF | D | 70.73 | 6.5 | 14.63 | 2.44 | 5.69 | 81.9 | 82.9 |
| 5pt | AC | RBF | MC-2 | 11.19 | 1.4 | 2.8 |  | 84.62 | 81.8 | 80 |
| 4pt | Control | RBF | MC-2 | 30.54 | 1.2 | 5.39 | 1.8 | 61.08 | 81.5 | 85 |
| 5pt | Control | RBF | U | 67.83 |  | 16.08 |  | 16.08 | 80.8 | 80.8 |
| 4pt | All | KNN-5 | D | 8.39 | 72.03 |  | 19.58 |  | 80.4 | 100 |
| 4pt | AC | RBF | MC-2 | 11.98 | 2.4 | 1.8 | 1.8 | 82.04 | 80 | 86.9 |
| 4pt | Control | RBF | U | 68.86 |  | 17.37 |  | 13.77 | 79.9 | 79.9 |
| 5pt | Raw | KNN-5 | D | 0.7 | 79.02 |  | 20.28 |  | 79.7 | 100 |
| 5pt | Raw | KNN-3 | D | 29.34 | 49.7 | 0.6 | 20.36 |  | 79 | 98 |
| 4pt | Raw | KNN-3 | D |  | 77.84 |  | 22.16 |  | 77.8 | 100 |
| 4pt | All | KNN-10 | D | 25.15 | 52.69 |  | 22.16 |  | 77.8 | N/A |
| 5pt | All | RBF | MC-3 | 4.9 |  | 1.4 |  | 93.71 | 77.8 | 77.8 |
| 5pt | Control | RBF | BM | 67.83 | 4.2 | 16.08 | 4.9 | 6.99 | 77.4 | 80.8 |
| 5pt | Control | RBF | D | 67.83 | 4.2 | 16.08 | 4.9 | 6.99 | 77.4 | 80.8 |
| 4pt | Raw | KNN-4 | D | 19.76 | 57.49 |  | 22.75 |  | 77.3 | 100 |
| 5pt | Raw | KNN-10 | D |  | 76.92 |  | 23.08 |  | 76.9 | N/A |
| 4pt | All | KNN-15 | D |  | 76.65 |  | 23.35 |  | 76.7 | N/A |
| 4pt | Raw | KNN-5 | D | 5.45 | 70.95 |  | 23.6 |  | 76.4 | 100 |
| 6pt | Control | RBF | MC-2 | 21.95 | 1.63 | 6.5 | 0.81 | 69.11 | 76.3 | 77.2 |
| 5pt | All | KNN-10 | D |  | 76.22 |  | 23.78 |  | 76.2 | N/A |
| 5pt | Control | RBF | MC-3 | 11.19 |  | 3.5 |  | 85.31 | 76.2 | 76.2 |
| 4pt | All | KNN-3 | D | 23.95 | 52.1 | 0.6 | 23.35 |  | 76.1 | 97.6 |
| 6pt | All | KNN-3 | D | 32.52 | 43.09 | 1.63 | 22.76 |  | 75.6 | 95.2 |
| 6pt | All | KNN-4 | D | 14.63 | 60.98 | 0.81 | 23.58 |  | 75.6 | 94.8 |
| 6pt | All | KNN-5 | D | 11.38 | 64.23 | 0.81 | 23.58 |  | 75.6 | 93.4 |
| 5pt | All | KNN-3 | D | 27.27 | 48.25 | 2.1 | 22.38 |  | 75.5 | 92.8 |
| 5pt | All | KNN-5 | D |  | 75.52 | 0.7 | 23.78 |  | 75.5 | 0 |
| 4pt | All | KNN-4 | D | 17.37 | 58.08 |  | 24.55 |  | 75.5 | 100 |
| 4pt | Control | RBF | D | 68.86 | 1.8 | 17.37 | 5.99 | 5.99 | 75.2 | 79.9 |
| 4pt | Raw | KNN-10 | D |  | 74.85 |  | 25.15 |  | 74.9 | N/A |
| 4pt | Control | RBF | BM | 68.86 | 1.2 | 17.37 | 6.59 | 5.99 | 74.5 | 79.9 |
| 4pt | Control | KNN-5 | D | 6.59 | 67.66 | 0.6 | 25.15 |  | 74.3 | 91.7 |
| 5pt | Raw | KNN-15 | D |  | 74.13 |  | 25.87 |  | 74.1 | N/A |
| 5pt | All | KNN-15 | D |  | 74.13 |  | 25.87 |  | 74.1 | N/A |
| 5pt | Raw | KNN-4 | D | 1.4 | 72.03 | 0.7 | 25.87 |  | 73.4 | 66.7 |
| 4pt | Control | KNN-4 | D | 8.98 | 63.47 | 1.2 | 26.35 |  | 72.5 | 88.2 |
| 5pt | All | KNN-4 | D | 12.59 | 59.44 | 0.7 | 27.27 |  | 72 | 94.7 |
| 4pt | Control | KNN-3 | D |  | 71.86 |  | 28.14 |  | 71.9 | 87.5 |
| 4pt | Raw | KNN-15 | D | 20.96 | 50.9 | 2.99 | 25.15 |  | 71.9 | N/A |
| 6pt | Raw | KNN-5 | D | 4.07 | 67.48 |  | 28.46 |  | 71.5 | 100 |
| 6pt | Raw | KNN-3 | D | 22.76 | 48.78 | 1.63 | 26.83 |  | 71.5 | 93.3 |
| 6pt | Raw | KNN-4 | D | 17.07 | 54.47 |  | 28.46 |  | 71.5 | 100 |
| 4pt | Control | KNN-15 | D |  | 69.46 |  | 30.54 |  | 69.5 | N/A |
| 6pt | Control | KNN-3 | D | 15.45 | 52.03 |  | 32.52 |  | 67.5 | 100 |
| 4pt | Control | KNN-15 | D |  | 67.07 |  | 32.93 |  | 67.1 | N/A |
| 5pt | AC | RBF | MC-3 | 2.8 |  | 1.4 |  | 95.8 | 66.7 | 66.7 |
| 6pt | All | KNN-10 | D | 2.44 | 61.79 |  | 35.77 |  | 64.2 | 100 |
| 6pt | Control | KNN-4 | D | 3.25 | 60.98 |  | 35.77 |  | 64.2 | 100 |
| 6pt | Raw | KNN-15 | D |  | 62.6 |  | 37.4 |  | 62.6 | N/A |
| 6pt | Control | KNN-15 | D |  | 62.6 |  | 37.4 |  | 62.6 | N/A |
| 6pt | All | KNN-15 | D |  | 62.6 |  | 37.4 |  | 62.6 | N/A |
| 5pt | AC | RBF | U | 31.47 |  | 18.88 |  | 49.65 | 62.5 | 62.5 |
| 5pt | Control | KNN-10 | D |  | 61.54 |  | 38.46 |  | 61.5 | N/A |
| 5pt | Control | KNN-3 | D | 11.89 | 48.95 | 4.2 | 34.97 |  | 60.8 | 73.9 |
| 5pt | Control | KNN-4 | D | 11.19 | 48.95 | 2.1 | 37.76 |  | 60.1 | 84.2 |
| 5pt | AC | RBF | D | 31.47 | 12.59 | 18.88 | 11.89 | 25.17 | 58.9 | 62.5 |
| 5pt | Control | KNN-5 | D | 7.69 | 51.05 |  | 41.26 |  | 58.7 | 100 |
| 5pt | AC | RBF | BM | 31.47 | 11.19 | 18.88 | 13.29 | 25.17 | 57 | 62.5 |
| 6pt | Control | KNN-5 | D |  | 56.91 |  | 43.09 |  | 56.9 | N/A |
| 6pt | Raw | KNN-15 | D |  | 56.1 |  | 43.9 |  | 56.1 | N/A |
| 5pt | Control | KNN-15 | D |  | 54.45 |  | 47.55 |  | 53.4 | N/A |
| 4pt | AC | RBF | U | 29.94 |  | 30.54 |  | 39.52 | 49.5 | 49.5 |
| 5pt | AC | KNN-5 | D | 1.4 | 46.85 |  | 51.75 |  | 48.3 | 100 |
| 4pt | AC | KNN-3 | D | 5.39 | 42.51 | 4.19 | 47.9 |  | 47.9 | 56.3 |
| 5pt | AC | KNN-4 | D | 4.2 | 42.66 |  | 53.15 |  | 46.9 | 100 |
| 5pt | AC | KNN-3 | D | 8.39 | 38.46 | 4.9 | 48.25 |  | 46.9 | 63.1 |
| 4pt | AC | RBF | BM | 29.94 | 5.39 | 30.54 | 9.58 | 24.55 | 46.8 | 49.5 |
| 4pt | AC | RBF | D | 29.94 | 5.39 | 30.54 | 9.58 | 24.55 | 46.8 | 49.5 |
| 4pt | AC | KNN-4 | D | 1.2 | 44.91 |  | 53.89 |  | 46.1 | 100 |
| 5pt | AC | KNN-10 | D |  | 45.45 |  | 54.55 |  | 45.5 | N/A |
| 4pt | AC | KNN-5 | D | 0.6 | 43.11 |  | 56.29 |  | 43.7 | 100 |
| 5pt | AC | KNN-15 | D |  | 42.66 |  | 57.34 |  | 42.7 | N/A |
| 4pt | AC | KNN-15 | D |  | 38.32 |  | 61.68 |  | 38.3 | N/A |
| 4pt | AC | KNN-15 | D |  | 34.13 |  | 65.87 |  | 34.1 | N/A |
| 6pt | AC | KNN-15 | D |  | 20.33 |  | 79.67 |  | 20.3 | N/A |
| 6pt | AC | KNN-5 | D |  | 19.51 |  | 80.49 |  | 19.5 | N/A |
| 6pt | AC | KNN-15 | D |  | 18.7 |  | 81.3 |  | 18.7 | N/A |
| 6pt | Control | KNN-15 | D |  | 18.7 |  | 81.3 |  | 18.7 | N/A |
| 6pt | AC | KNN-4 | D |  | 17.07 |  | 82.93 |  | 17.1 | N/A |
| 6pt | AC | RBF | BM | 5.69 | 3.25 | 38.21 | 13.82 | 39.02 | 14.7 | 13 |
| 6pt | AC | RBF | D | 5.69 | 2.44 | 38.21 | 14.63 | 39.02 | 13.3 | 13 |
| 6pt | AC | KNN-3 | D |  | 13.01 | 0.81 | 86.18 |  | 13 | 0 |
| 6pt | AC | RBF | U | 5.69 |  | 38.21 |  | 56.1 | 13 | 13 |
| 6pt | AC | RBF | MC-2 | Model Unsuccessful | | | | | N/A | N/A |
| 6pt | AC | RBF | MC-3 | Model Unsuccessful | | | | | N/A | N/A |
| 6pt | Control | RBF | MC-3 | Model Unsuccessful | | | | | N/A | N/A |
| 4pt | AC | RBF | MC-3 | Model Unsuccessful | | | | | N/A | N/A |
| 4pt | Control | RBF | MC-3 | Model Unsuccessful | | | | | N/A | N/A |


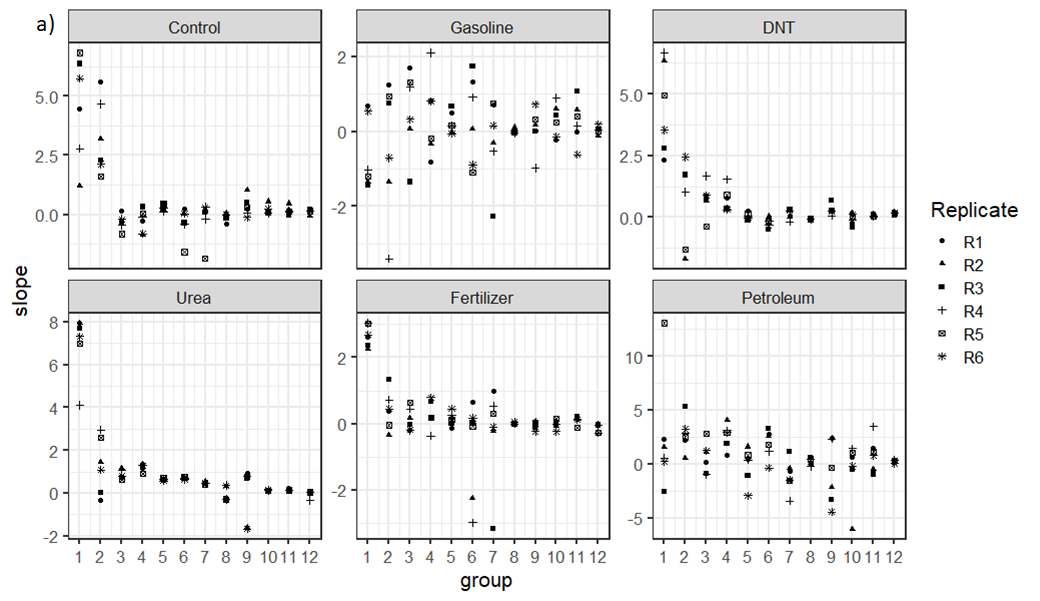


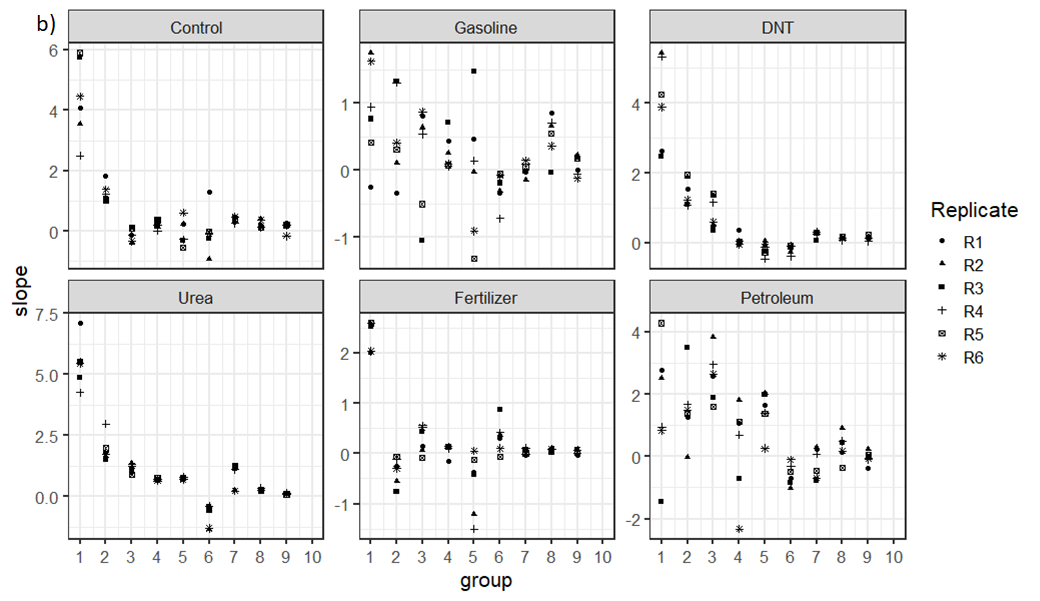


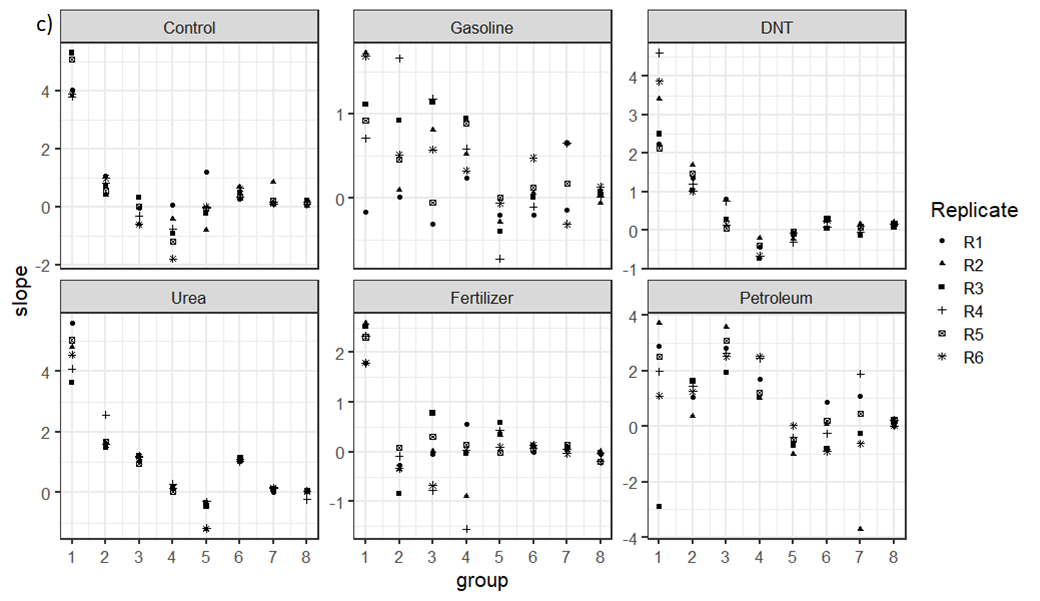

Supplement: S1 File — Values are listed in descending order by “General Accuracy”. All Values are percentages. Blank values imply 0%. AC is defined as standardization by anthropogenic compounds. RBF is defined as Radial Basis Function. KNN-# is defined as K-Nearest Neighbor Method where # is the K-value. For Interpreter, U is defined as “Unanimous”, BM as “Best Match”, D as “Dominant”, and MC-# is defined as the “Minimum Consensus” Interpreter where # is the minimum consensus. N/A means the model had no results for this category. Model Unsuccessful means there were insufficient correct classifications to train the model. To determine the observation window, data points were grouped in 4 (a), 5 (b), and 6 (c) point groups. X axis indicates group number i.e. the first group of four points, second group of four points, etc, Y axis is the slope of the four consecutive points, shape represents the replicate. (DOCX) [file pone.0260528.s001.docx]
